# Single-cell genomics: the human biomolecular and cell atlases

**DOI:** 10.1038/s41392-023-01676-1

**Published:** 2023-11-10

**Authors:** Yuqing Mei, Jingjing Wang, Guoji Guo

**Affiliations:** 1 Bone Marrow Transplantation Center of the First Affiliated Hospital, and Center for Stem Cell and Regenerative Medicine, Zhejiang University School of Medicine, Hangzhou, Zhejiang 310000 China; 2 Liangzhu Laboratory, Zhejiang University, Hangzhou, Zhejiang 311121 China; 3 Zhejiang Provincial Key Lab for Tissue Engineering and Regenerative Medicine, Dr. Li Dak Sum & Yip Yio Chin Center for Stem Cell and Regenerative Medicine, Hangzhou, Zhejiang 310058 China; 4 Institute of Hematology, Zhejiang University, Hangzhou, Zhejiang 310000 China

**Keywords:** Cell biology, Genetics

Recently, in a series of three papers published in *Nature*, the Human Biomolecular Atlas Program (HuBMAP) consortium reported three multi-model single-cell and spatial atlases.^1–3^ Each work covered more than a million cells and mapped them to specific locations in the body, providing new standards to study human organs in both health and disease states.

It’s worth noting that in 2020, colleagues from Zhejiang University in China developed the first human whole-organism cell atlas,^4^ permitting systematic analysis of human cell landscape at the single-cell level. As technologies continue to evolve, it is now possible to integrate additional layers of information into the atlas, such as spatial and temporal dynamics, epigenetic modifications, and protein expressions. The ongoing exploration of the human cell atlas holds immense promise for transforming healthcare, ushering in an era of precision medicine, and ultimately improving human well-being.

In 2022, the Human Cell Atlas (HCA) project published their monumental achievement in the field. This international collaborative effort, involving scientists from diverse disciplines, has successfully mapped the molecular landscape of the human body, providing unprecedented insights into the complexity of human system.

The Tabula Sapiens Consortium constructed a pan-organ human single-cell atlas, called Tabula Sapiens.^5^ They collected 500,000 live cells from multiple organ donors, depicting over 400 cell types across 24 tissues. They have revealed discoveries relating to shared behavior and subtle, organ-specific differences across cell types. At the same time, Cambridge teams have provided systematic insights into human immune cells.^6^ This study analyzed 330,000 immune cells from 16 tissues of 12 adult donors, revealing 100 distinct cell types and their distribution. Using a machine learning algorithm called CellTypist, researchers identified unique immune cell populations, and mapped their presence in different tissues. This comprehensive analysis shed light on the relationships between tissue resident immune cells and their microenvironments.

In these three recent papers from HuBMAP, Lake et al. constructed an extensive single-cell atlas spanning the corticomedullary axis of the kidney, covering the spectrum of conditions from healthy to acute kidney injury (AKI) and chronic kidney disease (CKD).^1^ The authors defined 51 major cell types, including rare and previously unidentified cell populations. Spatial mapping localized maladaptive tissue repair states that may impede renal tubule formation after injury and promote disease progression. The authors also identified cell-cell communication between maladaptive cells and adjacent stroma and inflammatory cells. Finally, the authors suggested that cell senescence may be an underlying mechanism leading to renal failure.

Hickey et al. conducted the pioneering in-depth spatial analysis in the intestine at the single-cell level.^2^ They used co-detection by indexing (CODEX) and single-nucleus RNA to map eight locations along the intestine using samples from nine individuals. This study revealed significant heterogeneity in cellular composition and organization along the intestine. The authors also identified novel subtypes of secretory cells. Notably, the authors highlighted that immune cells also exhibit spatial restriction and zonation, which was previously found in epithelial subtypes. By integrating spatial proteomics, single-cell RNA and ATAC technologies, this study has provided a valuable resource for understanding the human intestine.

Greenbaum et al. employed multiplexed ion beam imaging (MIBI) to build a spatiotemporal cell atlas of the maternal-fetal interface of from 66 individuals during the pre-pregnancy period (between 6 and 20 weeks).^3^ They found a remarkable shift in the immune compartment composition from maternal lymphoid to myeloid cells, along with an enrichment of tolerogenic subsets that tightly correlated with gestational age. Overall, this study explained how fetal cells invade and remodel blood vessels of maternal uterus and how maternal immune cells promote a tolerant environment between maternal uterine and fetal placental cells.

**Fig. 1:**
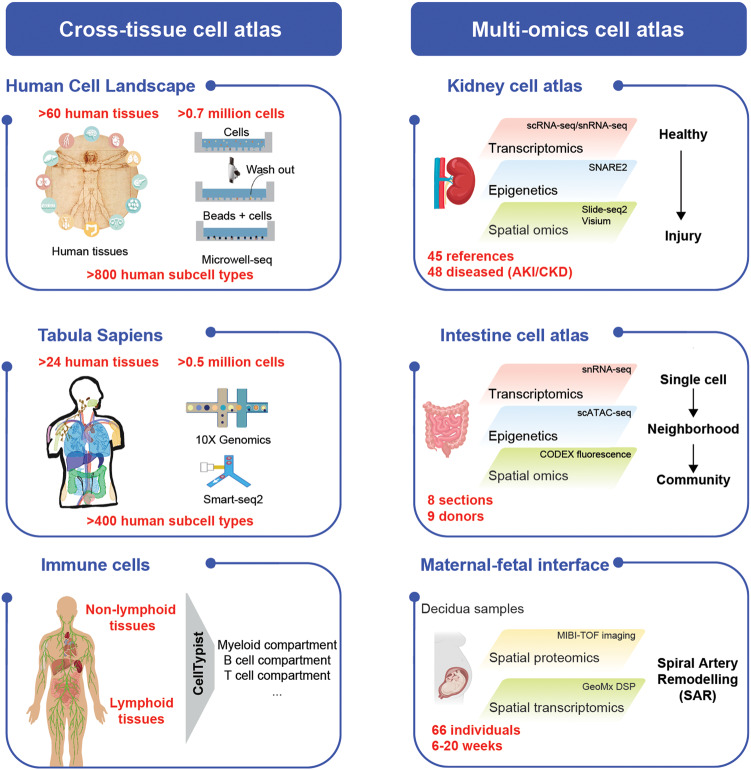
Single-cell atlases at different scales. Illustrations are the frameworks of representative single-cell landscapes, categorized into two groups based on different experimental sizes and methodologies

The exciting progress in Human Cell/Biomolecular Atlas represents a remarkable milestone in our quest to unravel the intricacies of human system (Fig. 1). As we move forward, the integration of single-cell and spatial technologies, functional genomics, computational analysis, and machine learning will continue to enhance our understanding of human biology. However, current research faces major challenges in integrating cross-platform, cross-laboratory, and cross-omics data. The batch effects from available technologies remains a problem. Future directions may include standardization of technical protocols and optimization of analytical pipelines. In addition, A more comprehensive cell atlas that covers disease cell stages is on the way. Finally, the application of Artificial intelligence (AI) for interpretation and modeling of complex atlas data may help to achieve predictive human biology at the single-cell level. A complete human cell map will help to advance disease diagnosis and personalized medicine.
